# Neuroanatomical Differences between First-Episode Psychosis Patients with and without Neurocognitive Deficit: A 3-Year Longitudinal Study

**DOI:** 10.3389/fpsyt.2013.00134

**Published:** 2013-10-17

**Authors:** Rosa Ayesa-Arriola, Roberto Roiz-Santiáñez, Rocío Pérez-Iglesias, Adele Ferro, Jesús Sainz, Benedicto Crespo-Facorro

**Affiliations:** ^1^Department of Psychiatry, School of Medicine, University of Cantabria, University Hospital Marqués de Valdecilla, IFIMAV, Santander, Spain; ^2^CIBERSAM, Centro Investigación Biomédica en Red Salud Mental, Madrid, Spain; ^3^Psychosis Studies Department, Institute of Psychiatry, London, UK; ^4^Department of Experimental Clinical Medicine, Inter-University Center for Behavioural Neurosciences (ICBN), University of Udine, Udine, Italy; ^5^CSIC, Spanish National Research Council, Institute of Biomedicine and Biotechnology of Cantabria, University of Cantabria, Santander, Spain

**Keywords:** first-episode psychosis, neuroanatomical, neurocognition, schizophrenia, cognitive deficit

## Abstract

**Background:** The course of cognitive function in first-episode psychosis (FEP) patients suggests that some individuals are normal or near normal whereas some cases present a marked decline. The goal of the present longitudinal study was to identify neuroanatomical differences between deficit and non-deficit patients.

**Methods:** Fifty nine FEP patients with neuroimage and neurocognitive information were studied at baseline and 3 year after illness onset. A global cognitive function score was used to classify deficit and non-deficit patients at baseline. Analysis of covariances and repeated-measures analysis were performed to evaluate differences in brain volumes. Age, premorbid IQ, and intracranial volume were used as covariates. We examined only volumes of whole brain, whole brain gray and white matter, cortical CSF and lateral ventricles, lobular volumes of gray and white matter, and subcortical (caudate nucleus and thalamus) regions.

**Results:** At illness onset 50.8% of patients presented global cognitive deficit. There were no significant differences between neuropsychological subgroups in any of the brain regions studied at baseline [all *F*(1, 54) ≤ 3.42; all *p* ≥ 0.07] and follow-up [all *F*(1, 54) ≤ 3.43; all *p* ≥ 0.07] time points. There was a significant time by group interaction for the parietal tissue volume [*F*(1, 54) = 4.97, *p* = 0.030] and the total gray matter volume [*F*(1, 54) = 4.31, *p* = 0.042], with the deficit group showing a greater volume decrease.

**Conclusion:** Our results did not confirm the presence of significant morphometric differences in the brain regions evaluated between cognitively impaired and cognitively preserved schizophrenia patients at the early stages of the illness. However, there were significant time by group interactions for the parietal tissue volume and the total gray matter volume during the 3-year follow-up period, which might indicate that cognitive deficit in schizophrenia would be associated with progressive brain volume loss.

## Introduction

Cognitive deficits are core features of schizophrenia that are already evident at early phases of the illness (1, 2), with more than 80% of patients showing deficits in one or more domains of cognitive function (3). There are, however, noticeable differences among patients, with a subgroup showing severe and debilitating cognitive dysfunctions, typical of Kraepelin’s description of dementia praecox (4), and other subgroup considered to be “neuropsychologically normal” (5). These distinguishable subgroups probably lie at different levels of severity on a continuum of causes or of different factors that might be influencing outcome (6).

Cognitive functioning has been associated with measures of brain structures both in schizophrenia patients and healthy subjects (7). It is now well established that schizophrenia is also associated with structural brain abnormalities (8, 9). Although some longitudinal magnetic resonance imaging (MRI) studies have reported a progressive brain tissue loss during the early years after the first psychotic episode of schizophrenia compared to healthy subjects (10–12), others have failed to reveal such progressive brain volume loss (13–18). Nevertheless, only few studies have examined those brain abnormalities that characterize the disorder in cognitive subgroups and, to the best of our knowledge, there are no previous studies that have examined progressive brain changes associated with cognitive deficit in schizophrenia.

The aim of this study was to identify neuroanatomical differences that possibly underlie neurocognitive function deficit in first-episode psychosis (FEP) patients followed up for 3-years. Previous studies have associated white matter abnormalities with cognitive deficit (19, 20). Perez-Iglesias and colleagues (19), using diffusion tensor imaging, showed that deficits in executive and motor functioning in patients with FEP were associated with reductions in white matter integrity, and Wexler et al. (20) found that neuropsychologically impaired patients had significantly smaller white matter volumes in several regions. On the basis of these studies, we hypothesized that patients with cognitive deficit would present a white matter volume diminution.

## Materials and Methods

### Study design and setting

Data for the present investigation were obtained from a large epidemiological and 3-year longitudinal intervention program of FEP (PAFIP) conducted at the outpatient clinic and the inpatient unit at the University Hospital Marques de Valdecilla, Santander, Spain. It conformed to international standards for research ethics and was approved by the local institutional review board. Informed consent of the participants was obtained after the nature of the procedures had been fully explained. The referrals to the PAFIP came from the inpatient unit and emergency room at the University Hospital Marques de Valdecilla, community mental health services and other community health care workers in Cantabria. A more detailed description of our program has been previously reported (21, 22).

### Subjects

All the patients included in PAFIP from February 2001 to December 2007 were invited to participate in this study. Patients referred to the program were selected if they met the following criteria: (1) 15–60 years; (2) living in the catchment area; (3) experiencing their first-episode of psychosis; (4) no prior treatment with antipsychotic medication or, if previously treated, a total life time of adequate antipsychotic treatment of less than 6 weeks; (5) DSM-IV criteria for brief psychotic disorder, schizophreniform disorder, schizophrenia, or schizoaffective disorder. Patients were excluded for any of the following reasons: (1) meeting DSM-IV criteria for drug dependence, (2) meeting DSM-IV criteria for mental retardation, (3) having a history of neurological disease or head injury. Our operational definition for a “first-episode of psychosis” included individuals with a non-affective psychosis (meeting the inclusion criteria defined above) who have not received previously antipsychotic treatment regardless the duration of psychosis. Individuals who entered the study received extensive clinical and psychopathological assessments and were examined by MRI scan. Only those patients who had a baseline neuropsychological assessment and completed both baseline and 3-year follow-up MRI scans were included in this study. Thus, 59 patients with a schizophrenia-spectrum disorder (schizophrenia *N* = 37, 62.7%; schizophreniform disorder, *N* = 15, 25.4%; schizoaffective disorder, *N* = 2, 3.4%; brief psychotic disorder, *N* = 2, 3.4%; not otherwise specified psychosis, *N* = 3, 5.1%) were included in the study.

The diagnoses were confirmed using the Structured Clinical Interview for DSM-IV (SCID-I) (23) carried out by an experienced psychiatrist 6 months after the baseline visit.

A group of healthy subjects (*N* = 43) who had no current or past history of psychiatric illness, including substance dependence, neurological or general medical disorders, as determined by using an abbreviated version of the Comprehensive Assessment of Symptoms and History (CASH) (24), was recruited from the local area. The controls underwent the same cognitive battery as the patients. After a detailed description of the study, each healthy subject gave their written informed consent to participate, in accordance with the local ethics committee (25).

There were no significant differences in a variety of variables (e.g., age at baseline, age of onset, academic level, alcohol, cannabis, or tobacco consumption, duration of untreated psychosis (DUP), duration of untreated illness (DUI), or symptomatology factors) between patients who were and those who were not included in the final analysis (all *p* > 0.236).

### Clinical assessment

Clinical symptoms were assessed by using the Brief Psychiatric Rating Scale total (BPRS) (26), the Scale for the Assessment of Negative Symptoms (SANS) (27), and the Scale for the Assessment of Positive Symptoms (SAPS) (28). We also divided psychopathology into three dimensions of symptoms: positive (scores for hallucinations and delusions), disorganized (scores for formal thought disorder, bizarre behavior, and inappropriate affect), and negative (scores for alogia, affective flattening, apathy, and anhedonia) (29).

### Premorbid and sociodemographic variables

Premorbid and sociodemographic information was recorded from patients, relatives, and medical records. Age of onset of psychosis was defined as the age when the emergence of the first continuous (present most of the time) psychotic symptom occurred. DUI was defined as the time from the first unspecific symptoms related to psychosis (for such a symptom to be considered, there should be no return to previous stable level of functioning) to initiation of adequate antipsychotic drug treatment. DUP was defined as the time from the first continuous (present most of the time) psychotic symptom to initiation of adequate antipsychotic drug treatment. Information about alcohol, cannabis, and tobacco consume were converted into binary variables coding for either the presence or absence of use.

### Medication assessment

The amount and type of medication being prescribed during the 3-year follow-up period was thoroughly recorded. Patients were randomized as part of an intervention program out of the scope of the present study. After written informed consent was obtained, patients were randomly assigned to Haloperidol (*N* = 8), Olanzapine (*N* = 12), Risperidone (*N* = 12), Quetiapine (*N* = 8), Ziprasidone (*N* = 10), and Aripiprazole (*N* = 9). At 3-year follow-up patients were on: Haloperidol (*N* = 3), Olanzapine (*N* = 8), Risperidone (*N* = 12), Quetiapine (*N* = 5) Ziprasidone (*N* = 4), Aripiprazole (*N* = 8), Amisulpride (*N* = 1), Clozapine (*N* = 2), and Risperidone depot (*N* = 4). Eight patients withdrew from the medication, 27 patients switched their medication during follow-up period, and 2 were taking more than one antipsychotic at the time of follow-up MRI. No reliable information on medication intake was available for four patients. Additional information about concomitant medications is available under request.

### Neuropsychological assessment

For the present study, baseline neuropsychological assessment was considered in both groups, patients, and normal control subjects. Baseline patients’ assessment was carried out when clinical status permitted in order to maximize cooperation, and occurred at a mean of 10.5 weeks after intake followed by assessment after 3 years. They were never assessed during a period of clinical exacerbation. A detailed description has been reported elsewhere (1).

For the analysis in this study a subset of measures was selected to assess eight major cognitive domains: (1) for measuring verbal memory we used the Rey Auditory Verbal Learning Test [RAVLT (30)], delayed recall; (2) for measuring visual memory we used Rey Complex Figure [RCF (31)], delayed reproduction; (3) for measuring executive functions we used Trail Making Test [TMT (32)], time to complete TMT-B; (4) for measuring working memory we used the Backward Digits scale [WAIS III (33)], total subscore; (5) for measuring speed of processing we used WAIS III subtest Digit Symbol, standard total score; (6) for motor dexterity we used Grooved Pegboard Handedness (34), time to complete with dominant hand; (7) for measuring attention we used Continuous Performance Test [CPT (35)], total number of correct responses; (8) The WAIS III subtest of Vocabulary was used as measure of premorbid IQ (34), standard total score.

In order to calculate a measure of Global Cognitive Functioning (GCF), raw cognitive scores were reversed when appropriate before standardization so they all have the same direction (the higher, the better). According to previous methodology (36), the GCF was calculated as *T*-scores (mean = 50, SD = 10) with raw scores of the healthy comparison sample. *T*-scores were converted to deficit scores that reflect presence and severity of cognitive deficit. Deficit scores on all tests were then averaged to create the GCF, which according to Keefe and colleagues deficit criterion (37), was dichotomized into two patients’ subgroups: “deficit” (GCF <1) and “non-deficit” (GCF ≥1) [see Ref. (6) for details].

### MRI data acquisition

A multimodal MRI protocol [T1, T2, and proton density (PD) sequences] was acquired at the University Hospital Marques de Valdecilla, Santander, Spain, using a 1.5-T General Electric SIGNA System (GE Medical Systems, Milwaukee, WI, USA). This multimodal approach was designed to optimize discrimination between gray matter, white matter, and cerebrospinal fluid. The T1-weighted images, using a spoiled grass (SPGR) sequence, were acquired in the coronal plane with the following parameters: echo time (TE) = 5 ms, repetition time (TR) = 24 ms, number of excitations (NEX) = 2, rotation angle = 45°, field of view (FOV) = 26 cm × 19.5 cm, slice thickness = 1.5 mm, and a matrix of 256 × 192. The PD and transverse relaxation time (T2)-weighted images were obtained with the following parameters: 3.0 mm thick coronal slices, TR = 3000 ms, TE = 36 ms (for PD) and 96 ms (for T2), NEX = 1, FOV = 26 cm × 26 cm, matrix = 256 × 192. The in-plane resolution was 1.016 mm × 1.016 mm. MRIs of patients and controls were evenly acquired during follow-up time.

### Image processing

Processing of the images after acquisition was done by using a family of software programs called BRAINS2 (38, 39). The T1-weighted images were spatially normalized and resampled to 1.0-mm^3^ voxels so that the anterior-posterior axis of the brain was realigned parallel to the anterior commissure/posterior commissure line and the interhemispheric fissure aligned on the other two axes. The T2- and PD-weighted images were then aligned to the spatially normalized T1-weighted image. These images were then subjected to a linear transformation into standardized stereotaxic Talairach atlas space to generate automated measurements of frontal, temporal, parietal, and occipital lobes and also the cerebellum and subcortical regions (39). To further classify tissue volumes into gray matter, white matter, and CSF, we used a discriminant analysis method of tissue segmentation based on automated training class selection that utilized data from the T1-weighted, T2-weighted, and PD sequences (40). The discriminant analysis method permits to identify the range of voxel intensity values that characterize GM, WM, and CSF. An 8 bit number is assigned to each voxel indicating its partial volume tissue content (10–70 for CSF, 70–190 for GM, and 190–250 for WM). In this study we examined the volumes of whole brain (WB), whole brain gray matter (WBGM), whole brain white matter (WBWM), cortical CSF (CCSF), and lateral ventricles (LV), gray and white matter volumes of cortical (occipital, parietal, temporal, and frontal lobes) and subcortical (caudate nucleus and thalamus) regions volume. Caudate and thalamus were delineated using a reliable and validated semiautomated artificial neural network (41). The procedure for measuring the volume of caudate and thalamus are explained in detail in previous studies (42, 43).

### Statistical analysis

The Statistical Package for Social Science, version 19.0 (SPSS Inc., Chicago, IL, USA), was used for statistical analyses. Significance was determined at the 0.05 level.

To examine brain volumetric differences between neurocognitive subgroups (no deficit vs. deficit) at baseline and 3-year follow-up, 1-way ANCOVA was performed. In each general linear model, the dependent measures were MRI volumes and the independent measure was group (no deficit vs. deficit). To test the hypothesis that the two groups would result in different progressive brain volume changes, repeated-measures analysis of covariance (repeated-measures ANCOVA) was performed for each ROI variable. The between-subject factor was group (no deficit vs. deficit) and the within subject factor was time (baseline and 3 year). Effects of time by group (interaction effect) were examined. Age, ICV, and premorbid IQ were included as covariates. There were no differences between groups related to age and ICV. However, there was a wide age range in our sample and the use of these two variables has been suggested in brain volume studies (44). The sample size (*n* = 59) provided sufficient power (>80%) to detect large effect sizes (*d*_Cohen_ > 0.8) but was underpowered (45%) to detect weak or modest effects (*d*_Cohen_ < 0.5).

Pearson correlation coefficients with age, ICV, and IQ as covariates were used to investigate possible statistical relationships between brain volume and GCF.

A prior directional hypothesis had been made for the brain measure analyses, thereby lessening the need for Bonferroni corrections. The analyses examining the relationships between brain measures and GCF were performed without prespecified hypotheses, and therefore Bonferroni adjustments were applied.

## Results

Demographic and clinical data are shown in Table 1. Neuropsychological baseline assessment showed that of the 59 patients included in the study, 30 (50.8%) presented general neurocognitive deficit. There were no statistically significant differences in relevant demographic and clinical characteristics between patients with neurocognitive deficit (*N* = 30) and patients without it (*N* = 29) at baseline (Table 1). However, the general neurocognitive deficit group showed a significant higher BPRS total score and greater severity of positive (SAPS total and positive dimension) and disorganized (scores for formal thought disorder, bizarre behavior, and inappropriate affect) symptoms at follow-up.

**Table 1:** **Sociodemographic and clinical characteristics of the two neurocognitive groups of patients**.

Characteristics	Non-deficit (*N* = 29)	Deficit (*N* = 30)	Statistics
Age at initial MRI, mean (SD), years	28.50 (6.93)	32.12 (9.97)	*F*(1, 57) = 2.59, *p* = 0.113
ICV, mean (SD)	1367.41 (128.78)	1343.19 (120.18)	*F*(1, 57) = 0.56, *p* = 0.458
Males, *N* (%)	18 (62.1)	20 (66.7)	χ^2^(1) = 1.36, *p* = 0.712
Time between scans, mean (SD), (range) days	1117.96 (39.58)	1120.63 (56.34)	*F*(1, 57) = 0.04, *p* = 0.835
Right-handed, *N* (%)	26 (89.7)	25 (83.3)	χ^2^(1) = 0.50, *p* = 0.478
Parental socioeconomic status, mean (SD)	3.45 (0.87)	3.60 (0.89)	*F*(1, 57) = 0.44, *p* = 0.512
Years of study, mean (SD)	10.89 (3.17)	10.13 (3.19)	*F*(1, 57) = 0.85, *p* = 0.360
Alcohol users, *N* (%)	17 (58.6)	19 (63.3)	χ^2^(1) = 1.38, *p* = 0.711
Cannabis users, *N* (%)	15 (51.7)	11 (36.7)	χ^2^(1) = 1.35, *p* = 0.244
Tobacco users, *N* (%)	17 (58.6)	16 (53.3)	χ^2^(1) = 0.17, *p* = 0.683
Age at onset, mean (SD), years	27.61 (6.28)	30.59 (9.79)	*F*(1, 57) = 1.91, *p* = 0.172
DUP mean, (SD), (median), months	10.74 (19.39)	18.35 (21.15)	*F*(1, 57) = 2.07, *p* = 0.155
DUI mean, (SD), (median), months	28.32 (32.79)	30.45 (29.54)	*F*(1, 57) = 0.07, *p* = 0.794
SANS at intake, mean (SD)	6.55 (4.95)	7.13 (5.37)	*F*(1, 57) = 0.19, *p* = 0.667
SANS at follow up, mean (SD)	1.89 (2.96)	3.34 (4.57)	*F*(1, 55) = 2.00, *p* = 0.162
SAPS at intake, mean (SD)	13.44 (4.44)	13.67 (4.17)	*F*(1, 57) = 0.04, *p* = 0.846
SAPS at follow up, mean (SD)	0.57 (1.40)	2.86 (4.62)	***F(1, 55) = 6.30, p = 0.015***
BPRS at intake, mean (SD)	62.00 (11.67)	63.50 (13.68)	*F*(1, 57) = 0.21, *p* = 0.653
BPRS at follow-up, mean (SD)	27.14 (4.33)	32.93 (14.17)	***F(1, 55) = 4.28, p = 0.043***
**SYMPTOM DIMENSIONS TOTAL SCORES AT BASELINE**			
Negative (mean) (SD)	4.75 (4.69)	5.40 (4.90)	*F*(1, 57) = 0.26, *p* = 0.610
Disorganized (mean) (SD)	6.07 (3.43)	6.10 (3.35)	*F*(1, 57) < 0.01, *p* = 0.972
Psychotic (mean) (SD)	7.37 (2.38)	7.57 (2.31)	*F*(1, 57) = 0.09, *p* = 0.760
**SYMPTOM DIMENSIONS TOTAL SCORES AT FOLLOW UP**			
Negative (mean) (SD)	1.54 (2.33)	2.90 (4.25)	*F*(1, 55) = 2.23, *p* = 0.141
Disorganized (mean) (SD)	0.25 (0.84)	1.45 (2.99)	***F(1, 55) = 4.16, p = 0.046***
Positive (mean) (SD)	0.32 (1.19)	1.41 (2.06)	***F(1, 55) = 5.95, p = 0.018***

BPRS, brief psychiatric rating scale; DUP, duration of untreated psychosis; DUI, duration of untreated illness; ICV, intracranial volume; SANS, scale for the assessment of negative symptoms; SAPS, scale for the assessment of positive symptoms; SD, standard deviation. Bold values were statistically significant (*p* < 0.05).

Neuropsychological data is presented in Table 2. The general neurocognitive deficit group had worse premorbid IQ, and showed consistently greater deficits all over cognitive domains. Worse executive function, poor motor dexterity, and particularly attentional deficits marked the more severely deficit patients.

**Table 2:** **Comparison of neurocognitive groups on neuropsychological variables (*Student’s t-*distribution with 58 degrees of freedom)**.

Characteristics	Non-deficit (*N* = 29)	Deficit (*N* = 30)	Statistics
Premorbid IQ	−0.08 (1.13)	−1.03 (1.19)	*t* = 3.07, *p* = 0.003
Attention	−0.39 (1.5)	−4.41 (5.31)	*t* = 3.98, *p* < 0.001
Verbal memory	−0.73 (1.08)	−1.31 (1.08)	*t* = 2.04, *p* = 0.046
Visual memory	−0.47 (1.1)	−1.16 (1.09)	*t* = 2.41, *p* = 0.019
Working memory	−0.23 (0.51)	−0.84 (0.75)	*t* = 3.66, *p* = 0.001
Executive function	−0.21 (0.74)	−2.51 (2.56)	*t* = 4.72, *p* = 0.001
Processing speed	−1.02 (1.15)	−2.02 (0.89)	*t* = 3.75, *p* = 0.001

Brain volumes at baseline in FEP subjects are presented in Table 3. There were no significant differences between neuropsychological subgroups in any of the brain regions studied at baseline [all *F*(1, 54) ≤ 3.42; all *p* ≥ 0.070] and follow-up [all *F*(1, 54) ≤ 3.43; all *p* ≥ 0.07] time points (Table 3). Patients with cognitive deficit showed overall lower gray and white matter volumes but these differences did not reach statistical significance.

**Table 3:** **Comparison of MRI brain volumes between deficit first-episode psychosis (FEP) and non-deficit FEP patients at baseline and follow-up and the group by time interaction**.

	Basal							Follow-up								
	No deficit (*N* = 29)		Deficit (*N* = 30)					No deficit (*N* = 29)		Deficit (*N* = 30)					Group × time	
	Mean	SD	Mean	SD	% Diff	*F*(1, 54)	*p*	Mean	SD	Mean	SD	% Diff	*F*(1, 54)	*p*	*F*(1, 54)	*p*
Total brain tissue	1286.67	119.47	1254.77	118.32	2.5	1.41	0.240	1279.72	121.67	1234.12	126.45	3.6	3.43	0.070	2.07	0.156
Frontal lobe tissue	430.55	38.56	420.36	37.69	2.4	0.48	0.491	425.51	41.05	412.32	44.88	3.1	0.37	0.543	0.00	0.958
Parietal lobe tissue	249.22	25.11	246.06	26.62	1.3	0.14	0.710	248.9	22.44	241.94	27.67	2.8	0.55	0.463	**4.97**	**0.030**
Temporal lobe tissue	227.87	25.25	219.23	22.37	3.8	1. 51	0.225	225.39	26.89	215.74	23.09	4.3	2.03	0.160	0.28	0.599
Occipital lobe tissue	121.17	16.74	120.6	17.72	0.5	0.34	0.560	122.92	15.42	118.56	17.42	3.5	0.09	0.768	2.06	0.157
Total brain GM	779.42	72.43	759.18	87.63	2.6	0.52	0.473	768.1	66.92	738.61	85.42	3.8	0.41	0.523	**4.32**	**0.042**
Frontal lobe GM	261.06	21.3	254	28.21	2.7	0.02	0.900	259.11	23.29	247.52	30.43	4.5	0.47	0.494	1.11	0.297
Parietal lobe GM	137.43	15.61	135.92	19.78	1.1	1. 58	0.214	135.82	13.62	131.81	18.55	3.0	0.04	0.837	3.72	0.059
Temporal lobe GM	156.56	17.56	150.69	17.68	3.7	0.00	0.956	153.99	18.21	146.8	17.84	4.7	0.63	0.431	1.82	0.183
Occipital lobe GM	63.2	9.84	63.44	10.33	-0.4	1.73	0.194	63.39	9.81	61.00	9.10	3.8	0.00	0.994	2.19	0.145
Total brain WM	507.25	60.77	495.59	50.32	2.3	2.07	0.156	511.62	65.09	495.51	55.25	3.1	2.09	0.154	0.02	0.878
Frontal lobe WM	169.49	21.67	166.37	18.31	1.8	1.06	0.308	166.4	21.36	164.8	20.15	1.0	0.18	0.677	0.15	0.230
Parietal lobe WM	111.79	14.02	110.13	13.15	1.5	0.90	0.346	113.08	11.82	110.13	14.44	2.6	1.67	0.202	0.24	0.623
Temporal lobe WM	71.31	11.89	68.55	8.91	3.9	3.42	0.070	71.39	12.56	68.94	8.44	3.4	1.77	0.189	1.43	0.237
Occipital lobe WM	57.97	9.74	57.17	10.14	1.4	0.12	0.727	59.53	8.71	57.56	10.57	3.3	0.34	0.563	0.20	0.657
Cortical CSF	44.84	18.02	50.44	22.5	-12.5	1.16	0.287	46.86	18.48	57.76	32.89	-23.3	2.52	0.118	0.71	0.404
Lateral ventricles	14.73	6.84	14.14	6.15	4.0	0.46	0.501	14.82	7.81	14.79	7.47	0.2	0.15	0.703	0.38	0.543
Thalamus	11.76	1.5	11.66	1.3	0.9	2.02	0.161	11.46	1.46	11.29	1.24	1.5	1.02	0.317	0.03	0.866
Caudate	5.37	0.76	5.25	0.75	2.2	0.22	0.642	5.24	0.75	5.11	0.77	2.5	0.10	0.750	0.52	0.476

CSF, cerebrospinal fluid; Diff, difference; GM, gray matter; WM, white matter, percent difference was calculated as 100 × (VolDeficit − VolNoDeficit)/VolNoDeficit. Bold values were statistically significant (*p* < 0.05).

**Figure 1:**
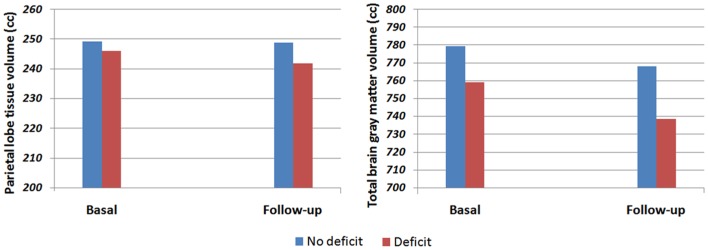
**Total gray matter and parietal tissue volume in deficit and non-deficit first-episode psychosis patients**.

There was a significant time by group interaction for the parietal tissue volume [*F*(1, 54) = 4.97, *p* = 0.030], with the general neurocognitive deficit group showing a greater volume decrease (1.67%) than the non-deficit group (0.13%). Similarly, there was also a significant time by group interaction for the total gray matter volume [*F*(1, 54) = 4.31, *p* = 0.042], showing a greater reduction in the general neurocognitive deficit group (2.71%) than in the non-deficit group (1.45%) (see Figure 1). Interestingly, when the analyses were controlled by possible confounding variables (sex, DUP, tobacco, cannabis, and alcohol consumption) only the parietal lobe tissue showed a significant group by time interaction.

No significant correlations between brain volume at baseline and GCF were found. At follow-up period, there were significant negative correlations between GCF and parietal tissue lobe (*r* = −0.29, *p* = 0.031) and temporal lobe gray matter (*r* = −0.27; *p* = 0.049). However, these correlations were weak and did not remain significant after correcting for multiple testing (Bonferroni correction).

## Discussion

In the present study, a GCF index was calculated to identify schizophrenia patients who had general neurocognitive deficit at baseline (6). Contrary to our expectations, there were no brain volume differences between the cognitively impaired and cognitively preserved groups at any of the time-point studied. However, there were significant time by group interactions for the parietal tissue volume and the total gray matter volume, with the general neurocognitive deficit group showing a greater reduction in both regions during the 3-year follow-up interval. This is, to the best of our knowledge, the first study to examine progressive brain changes in schizophrenic cognitive deficit.

Neuropsychological assessment carried out at baseline indicated that 50.8% of the patients included in the study presented general neurocognitive deficit. These results are in full agreement with previous studies in FEP patients (2).

Only two previous cross-sectional studies (20, 45) have examined brain volume differences between cognitive subgroups in schizophrenia and their results have been inconclusive. Supporting our results, Ortiz-Gil and colleagues (45) did not find differences in lateral ventricular volume or WB volume between cognitively intact and cognitively deficit schizophrenia patients. Using Voxel-based morphometry, they also failed to detect significant difference in volumes of gray and white matter between those groups. However, Wexler et al. (20), using VBM to compare neuropsychology near normal and neuropsychology impaired subgroups, found that these groups differed significantly from each other in white matter volumes of the sensorimotor and parietal-occipital regions, with the neuropsychology impaired group showing smaller volumes in these brain regions. Nonetheless, it is of note that this VBM study did not adopt any statistical procedures to control for multiple comparisons. It is important to take into account that mean duration of illness among patients was above 18 years in both studies, while our patients had a shorter duration of illness (non-deficit: 28.32 months, deficit: 30.45 months).

Only cross-sectional studies have addressed the relationship between cognitive deficit and brain structure in schizophrenia. However, it has been suggested that this relationship may not be adequately assessed in a cross-sectional study (45). We found significant time by group interactions for the parietal tissue volume and the total gray matter volume, with the general neurocognitive deficit group showing a greater reduction in both regions (parietal tissue volume and the total gray matter volume) during the 3-year follow-up interval. In a recent study (13) we found that brain tissue volumes decrease in patients at early years after the first episode was similar to that found in healthy controls. Although several longitudinal studies in schizophrenia have described a greater degree of brain tissue volumes decrease in the early stage of the illness (10–12), others have failed to confirm these findings (13–18). For a review see Olabi et al. (46). Taken this together, we might speculate that the progressive brain volume loss found in schizophrenia might be associated with this general cognitive deficit patients’ subgroup.

### Limitations

A uniform follow-up interval using the same MRI scanner and protocol, and a thorough clinical investigation during the follow-up period add strength to the conclusions drawn from this study. However, several limitations should be taken into account when interpreting the results of the current study. First, the diagnostic heterogeneity of the sample might bias our findings. Second, and given the fact that schizophrenia is a life-long disease, a follow-up period of 3 years may be too short to demonstrate other subtle changes. Third, analyses taking into account if neurocognitive function maintained, declined, or improved during follow-up could not be conducted because of the small sample size in our study. Fourth, a major confounding factor could be the intake of antipsychotic medication (47). Some studies have showed a relationship between antipsychotic medication use and longitudinal brain volume change in schizophrenia (10, 48), although others have failed to clearly demonstrate an influence of antipsychotic medication on brain volume change (49–51). Some patients withdrew from their medication, and most of them switched medication during the 3-year follow-up period, which makes the investigation of the effects of different types of antipsychotics an unfeasible study. Fifth, this study only measured a number of structures, and finally, brain volume changes in schizophrenia are subtle, so the sample size might be considered small to make any definitive assertions. While our data provided sufficient power to detect large effects, the detection of weak effects requires large study populations.

## Conclusion

In conclusion, our results, based on a representative sample of first-episode schizophrenia-spectrum patients, do not confirm the presence of significant morphometric differences between cognitively impaired and cognitively preserved schizophrenia patients at the early stages of the illness. However, there were significant time by group interactions for the parietal tissue volume and the total gray matter volume during the 3-year follow-up period, which might indicate that cognitive deficit in schizophrenia would be associated with progressive brain volume loss. Further investigations are warranted to fully clarify the relationship between cognitive deficit and brain structure in schizophrenia.

## Conflict of Interest Statement

The study, designed and directed by Benedicto Crespo-Facorro, conformed to international standards for research ethics and was approved by the local institutional review board. Unrestricted educational and research grants from AstraZeneca, Pfizer, Bristol-Myers Squibb, and Johnson & Johnson provided support to PAFIP activities. No pharmaceutical industry has participated in the study concept and design, data collection, analysis and interpretation of the results, and drafting the manuscript. Prof. Crespo-Facorro has received unrestricted research funding from AstraZeneca, Pfizer, Bristol-Myers Squibb, and Johnson & Johnson that was deposited into research accounts at the University of Cantabria. Prof. Crespo-Facorro has received honoraria for his participation as a speaker at educational events from Pfizer, Bristol-Myers Squibb, and Johnson & Johnson and consultant fees from Pfizer. The rest of the authors report no additional financial.
